# Multi-task learning for cross-platform siRNA efficacy prediction: an in-silico study

**DOI:** 10.1186/1471-2105-11-181

**Published:** 2010-04-10

**Authors:** Qi Liu, Qian Xu, Vincent W Zheng, Hong Xue, Zhiwei Cao, Qiang Yang

**Affiliations:** 1 College of Life Science and Biotechnology, Tongji University, China; 2 Department of Computer Science and Engineering, Hong Kong University of Science and Technology, Hong Kong; 3 Department of Biochemistry, Hong Kong University of Science and Technology, Hong Kong; 4 Shanghai Center for Bioinformation Technology, China

## Abstract

**Background:**

Gene silencing using exogenous small interfering RNAs (siRNAs) is now a widespread molecular tool for gene functional study and new-drug target identification. The key mechanism in this technique is to design efficient siRNAs that incorporated into the RNA-induced silencing complexes (RISC) to bind and interact with the mRNA targets to repress their translations to proteins. Although considerable progress has been made in the computational analysis of siRNA binding efficacy, few joint analysis of different RNAi experiments conducted under different experimental scenarios has been done in research so far, while the joint analysis is an important issue in cross-platform siRNA efficacy prediction. A collective analysis of RNAi mechanisms for different datasets and experimental conditions can often provide new clues on the design of potent siRNAs.

**Results:**

An elegant multi-task learning paradigm for cross-platform siRNA efficacy prediction is proposed. Experimental studies were performed on a large dataset of siRNA sequences which encompass several RNAi experiments recently conducted by different research groups. By using our multi-task learning method, the synergy among different experiments is exploited and an efficient multi-task predictor for siRNA efficacy prediction is obtained. The 19 most popular biological features for siRNA according to their jointly importance in multi-task learning were ranked. Furthermore, the hypothesis is validated out that the siRNA binding efficacy on different messenger RNAs(mRNAs) have different conditional distribution, thus the multi-task learning can be conducted by viewing tasks at an "mRNA"-level rather than at the "experiment"-level. Such distribution diversity derived from siRNAs bound to different mRNAs help indicate that the properties of target mRNA have important implications on the siRNA binding efficacy.

**Conclusions:**

The knowledge gained from our study provides useful insights on how to analyze various cross-platform RNAi data for uncovering of their complex mechanism.

## Background

RNA interference (RNAi) is the process through which a double-stranded RNA (dsRNA) induces gene expression silencing, by either degradation of sequence-specific complementary mRNA or repression of translation [1]. Nowadays, RNAi has become an effective tool to inhibit gene expression, serving as a potential therapeutic strategy in viral diseases, drug target discovery and cancer therapy [2]. The key inhibition mechanism of RNAi is triggered by introducing a short interfering double-stranded RNA (siRNA,19~ 27 bp) into the cytoplasm, where the guide strand of siRNA (usually antisense strand) is incorporated into the RNA-induced silencing complex (RISC) that binds to its target mRNA and the expression of the target gene is blocked. How to design siRNAs with high efficacy and high specificity for their target genes is one of the critical research issues [3-7].

So far, considerable progress has been made in studying the silencing capacity of siRNAs (the siRNA binding efficacy). Some fundamental empirical guidelines for designing efficient siRNA molecules have been presented [8,9]. Further investigations include the study of the RNAi mechanism itself as well as characteristics of siRNAs with either high or low silencing capacity [10-16]. In total, these studies have led to several advanced algorithms and tools that allow the selection of potent siRNAs or the prediction of the efficacy of siRNA for gene silencing [13,17-26].

Computational models for siRNA efficacy prediction are often constructed in a training phase. The training data consist of a collection siRNA sequences and related inhibiting efficacy vis-a-vis their target genes. In the testing phase, trained models are applied to new instances, when potential characteristics related to siRNA efficacy are extracted from siRNA sequences or target mRNA and used for the prediction of siRNAs efficacy for new targets. This procedure is generally formulated as a classification or regression model [24]. Although various statistical and machine learning methods have been proposed in the last few years [24,27,28], there is limited success in predicting siRNA efficacy due to the diversity of data and limited sizes of available siRNA datasets. The problem caused by the differences in the training data pose difficulties for in-silico siRNA design. Typically, the RNAi data are provided by different research groups under different platforms/protocols in different experimental scenarios. This kind of data is refereed as "cross-platform" to emphasize the considerable diversity in such data. We observed that usually the observations (siRNA efficacy) from multiple platforms may not have an identical conditional distribution (i.e. the same residual variance) due to: First, a variety of assays/platforms/scales exist for measurements of the siRNA efficacy, such as different cell types (Hela, fibroblasts), test methods (Western Blotting, real-time PCR) or siRNA delivery methods (vectors method, synthetic oligos method). Second, there may exist very different concentrations of siRNAs used in different experiments. Finally, large differences can be found in sub-optimal time intervals between transfection and down-regulation measurement etc [24,29].

As we show later in the experimental part, a naive integration of the data for siRNA efficacy prediction will only result in poor performance. This data distribution diversity problem has largely been ignored in many previous studies, such as the P*ȧ*l Sætrom data [24], a classical dataset for siRNA efficacy prediction. This dataset has been used as a benchmark for training and testing in several computational studies for siRNA efficacy prediction, but the issue of non-identical conditional distribution has not received sufficient attention [30,31].

Since different RNAi experiments encompass siRNAs that are partially targeted on different mRNAs, how to jointly utilize different experimental datasets becomes a critical issue for large-scale RNAi screening analysis. Solutions to this problem are expected to provide new insights into the RNAi mechanism in a large-scale view. In our study, although cross-platform siRNA datasets may have different conditional distribution of their efficacy, they are related to a common biological problem and can be viewed as different prediction tasks under the same latent variables. This observation inspires us to exploit the possible synergies between different datasets, rather than combining them directly, to learn a *multi-task predictor *jointly and simultaneously for siRNA efficacy prediction. This predictor will allow different classification tasks to enhance each other during the training process, which eventually makes the efficacy prediction better than when the datasets are naively combined, or when the datasets are used separately.

In this paper, the cross-platform model construction issue was addressed by applying a simple, yet effective linear regression model based on the multi-task learning paradigm. This model was applied on multiple datasets for siRNA efficacy prediction. Recently, [32] presented a multi-task learning approach to learning drug combinations for drug design. In [33], a multi-task classification approach is applied on multiple platforms for finding out a small number of highly significant marker genes to aid in biological studies, where the emphasis is on feature selection across platforms. In [34], a novel transfer learning technique is applied to address such cross-platform siRNA efficacy prediction problem where the focus is on using the auxiliary domains to help improve the regression performance of a target class. To the best of our knowledge, our work is one of the first to apply the multi-task learning model for siRNA efficacy analysis for learning regression models.

To test our multi-task regression learning framework, extensive experiments were conducted to show that multi-task learning is naturally suitable for cross-platform siRNA efficacy prediction. The biological features were ranked to derive the most important common features for siRNA design across different experiments on this model. Furthermore, our experiments also validate the observation that the siRNA efficacy depends on the properties of the targeted mRNA, instead of merely on the properties of siRNA sequence. We also conjecture that continued computational siRNA efficacy study can be benefited greatly from the multi-task learning framework by focusing on a much smaller task level, where we can take, for example, each mRNA and its binding siRNAs as a task, rather than an entire experiment as a task.

## Methods

### Data source

Our study was performed on the siRNA efficacy dataset compiled by Shabalina et al., which contains 653 19-nt siRNAs targeted on 52 genes (no homology genes between them) from 14 cross-platform experiments [23]. The general description of this data source is given in Table 1, from which we can see that different experiments actually have different output label spaces in the evaluation of siRNA efficacy. It is reported that this is a mixture set of dataset including a broad range of siRNA concentrations, which, in distribution, is substantially biased towards the high end (over 300 siRNAs tested at 100 nM concentrations) in the evaluation of siRNA efficacy. The diversity in the data explains partly why the different measurement errors are non-trivial [23] [Additional file 1]. In addition, another two experiments with 32 siRNAs targeting on 10 distinct mRNAs are included in our study as two independent test sets [23]. The siRNA efficacy in these experiments was tested at very low concentrations to show that the effectiveness of our multi-task learning paradigm for predicting the efficacy of siRNAs is independent on concentrations.

**Table 1:** Description of the 14 cross-platform RNAi experiments as well as another 2 independent experiments performed at low siRNA concentrations.

Experiments	#mRNA	#siRNA	Platform label scale (min-max)
E1	2	179	4.0-127.8

E2	2	67	22.0-118.8

E3	1	14	2-52

E4	10	50	1.0-115.7

E5	2	12	18-110

E6	4	50	5.8-124.4

E7	3	19	20-127

E8	21	103	16.0-100.0

E9	1	34	1.5-93.9

E10	1	6	32-77

E11	2	24	5-120

E12	2	20	11.4-76.4

E13	1	5	0-34

E14	3	40	14-110

IE1	6	20	1.56-100

IE2	4	12	1-80

"E" denotes "Experiment";"IE" denotes "Independent experiment".

In our study, the same 19 parameter values were adopted for siRNA efficacy prediction as presented by Shabalina et al. [23] (see Table 2), since these parameters have covered most of the reported features that are significantly correlated with siRNA efficacy so far, such as nucleotide content of G, nucleotide content of U and position-dependent nucleotide etc. Under our multi-task learning paradigm, a quantitative evaluation of these 19 features will be provided to reveal the relevance of these 19 features to siRNA design, as shown in the next section.

**Table 2:** Feature weights for siRNA design derived from multi-task learning

**No**.	Feature	Weight
1	position-dependent nucleotide consensus: sum	0.1954
2	Δ G difference between positions 1 and 18	0.0987
3	Δ G of sense-antisense siRNA duplexes	0.0774
4	position-dependent nucleotide consensus: preferred	0.0733
5	preferred dinucleotide content index	0.0726
6	local target mRNA stabilities (Δ G)	0.0651
7	position-dependent nucleotide consensus: avoided	0.0640
8	nucleotide content: U	0.0603
9	stability (Δ G) of dimers of siRNAs antisense strands	0.0537
10	stability profile for each two neighboring base pairs in the siRNA sense-antisense in position 1	0.0384
11	siRNA antisense strand intra-molecular structure stability (Δ G)	0.0327
12	avoid dinucleotide content index	0.0324
13	stability profile for each two neighboring base pairs in the siRNA sense-antisense in position 13	0.0298
14	stability profile for each two neighboring base pairs in the siRNA sense-antisense in position 18	0.0279
15	nucleotide content: G	0.0267
16	stability profile for each two neighboring base pairs in the siRNA sense-antisense in position 2	0.0222
17	stability profile for each two neighboring base pairs in the siRNA sense-antisense in position 6	0.0159
18	stability profile for each two neighboring base pairs in the siRNA sense-antisense in position 14	0.0138
19	frequency of potential targets for siRNA	0.0000

We should explain the reasons for why this particular data source is chosen: First, the data source contains nearly all the RNAi experiments with numerical siRNA efficacy values reported in recent studies, thus proven to be a complete dataset for training regression models for siRNA efficacy prediction. Second, the data source is a mixture dataset with cross-platform experiments stated in P*ȧ*l Sæ trom dataset, a dataset misused by several computational siRNA efficacy prediction models where its data diversity is not considered [30,31]. We want to use the multi-task learning paradigm to address this cross-platform issue by comparing our test results with those of traditional studies. We noted that in the current study, we only focused on the regression model rather than the general classification models, since the siRNA efficacy values are in nature continuously valued under different experimental platforms and we don't want to waste any data information in using our model. Though our model is designed for regression problem, it's actually also suitable for the classification problem with categorical data as input. To support our argument, we applied our model in multi-task classification with the siRecords dataset [22], which normally standardized siRNA with consistent efficacy ratings across different platforms. The results are listed in the supplementary materials [Additional file 1], and they also indicate that our multi-task classification model is significantly better the single-task classification models.

### Linear ridge regression model

Given a representation of siRNAs as feature vectors, a linear ridge regression model was applied [35] to predict the novel siRNA efficacy from a set of siRNAs with known efficacy. Linear ridge regression is a classical statistical technique that aims to find a linear function that models the dependencies between covariances  in ℝ^*d *^and response variables  in ℝ, where *d *is the number of data features. The standard way to handle this problem is using the ordinary least square (OLS) method, which minimizes the squared loss:(1)

However, due to limited training examples, the variance of the estimated *w *by OLS may be large, and thus the estimation is not reliable. An effective way to overcome this problem is to penalize the norm of *w *as in ridge regression. Instead of minimizing squared errors, ridge regression minimizes the following cost:(2)

where λ is a fixed positive number. By introducing the regularization parameter λ, the ridge regression can reduce the estimated variance at the expense of increasing training errors. The regularization parameter λ controls the trade-off between the bias and variance of the estimate. In the linear ridge regression model, it is shown that the predicted label (i.e., *w*^*T *^*x*) of a new unlabeled example *x *is:(3)

where *K *is the matrix of dot products of the vectors {*x*_*i*_, *i *= 1,2, ..., *n*} in the training set:(4)

and κ is the vector of dot products of *x *and the vectors in the training set:(5)

It should be noted that this model could be generalized to kernel ridge regression by using the kernel trick [36]. However, model selection is not our main focus here. Various regression models can be applied, but we choose the linear ridge regression as our regression model based on the following reasons: (1) The performance of linear ridge regression model is comparable to most of the state-of-art regression models on siRNA efficacy prediction, and it is simple enough in representation [29]. We applied the sophisticated support vector regression (SVR) with both linear kernel and radial basis function kernel in siRNA efficacy prediction, and we obtained nearly the same (even worse) prediction results as compared to linear ridge regression (See Results and Discussion). (2) We also want to exploit the feature importance across the platforms for better siRNA design. This goal cannot be achieved if we use a kernel regression model since it will map the input features as some non-meaningful high-dimensional representations.

In our experimental study, 5-fold cross-validation was applied to find the optimal regularization parameter that minimizes the cross-validation errors. For all the 14 experiments, 5-fold cross-validation is performed on 5 regularization parameter regions respectively, i.e. [0.001,0.1] with interval 0.001, [0.01,0.1] with interval 0.01, [0.1,1] with interval 0.1, [1,10] with interval 1 and [10,100] with interval 10. Finally λ = 10 was obtained by evaluation of the total cross-validation errors in the 14 experiments. This parameter was kept the same throughout our study for consistent comparison.

### Performance Measurement

In our experiments, the proposed multi-task learning and traditional single task learning were evaluated based on root mean squared error (RMSE) [35], which is usually used as a measurement of the prediction ability in the regression model. The residual *e *is the difference between the observed data and the fitted model, denoted as:(6)

where *y*_*i *_is the observed siRNA efficacy and  is the predicted siRNA efficacy. The root mean squared error is defined as follows:(7)

where *n *is the number of predicted siRNA sequences. The smaller the RMSE is, the better the predict performance is.

### Paired *t*-test for model comparison

In our study, the paired *t*-test and *F*-test is performed to compare multi-task learning versus single-task learning in siRNA efficacy prediction [37]. Paired *t*-test is proven to work well by machine learning community in measuring the significance of one model outperforming another model and it is suitable for the most common data distribution assumption (say, normal distribution, instead of specific chi-squared distribution, for example) when we don't know the exact data distribution. To be briefly, this test is trying to determine whether the mean of a set of samples, i.e., the cross-validation estimates for the various datasets (tasks) is significantly greater than, or significantly less than the mean of another, followed by the assumptions that the observed data are from a matched subject and are drawn from a population with nearly to normal distribution.

More specifically, given two paired sets *X*_*i *_and *Y*_*i *_of *n *measured values, which could be the error rates evaluated by RMSE for each experiments under the single-task learning model and multi-task learning model in out study, the paired *t*-test determines whether this two model differ from each other in a significant way under the assumptions that the paired prediction error rate differences for each experiment are independent and identically normally distributed.

To apply the paired *t*-test, let:(8)

Then define *t *by:(10)

where *n- *1 is the statistic degrees of freedom. Once a *t *value is determined, a *p*-value can be found using a table of values from Student's *t*-distribution to determine the significance level at which two models differ.

### Multi-task learning for siRNA efficacy prediction

#### Computational framework

Multi-task learning has been developed in machine learning research to situations where multiple related learning tasks are accomplished together [38-46]. It has been proven to be more effective than learning each task independently when there are explicit or hidden inter-relationship among the tasks that can be exploited [47]. The intuition underlying the framework is that the multiple related tasks can benefit each other by sharing the data and features across the tasks, which can often boost the learning performance of each single task. Such an advantage is especially evident when the number of labeled data in each task is limited, such that training on each single task with insufficient labeled data may not work well. Recently, researchers have begun to resort to the multi-task learning model to solve biological problems, such as medical diagnosis, tumor classification and drug screening [48-50]. However, applications of multitask learning in bioinformatics have just begun.

**Figure 1:**
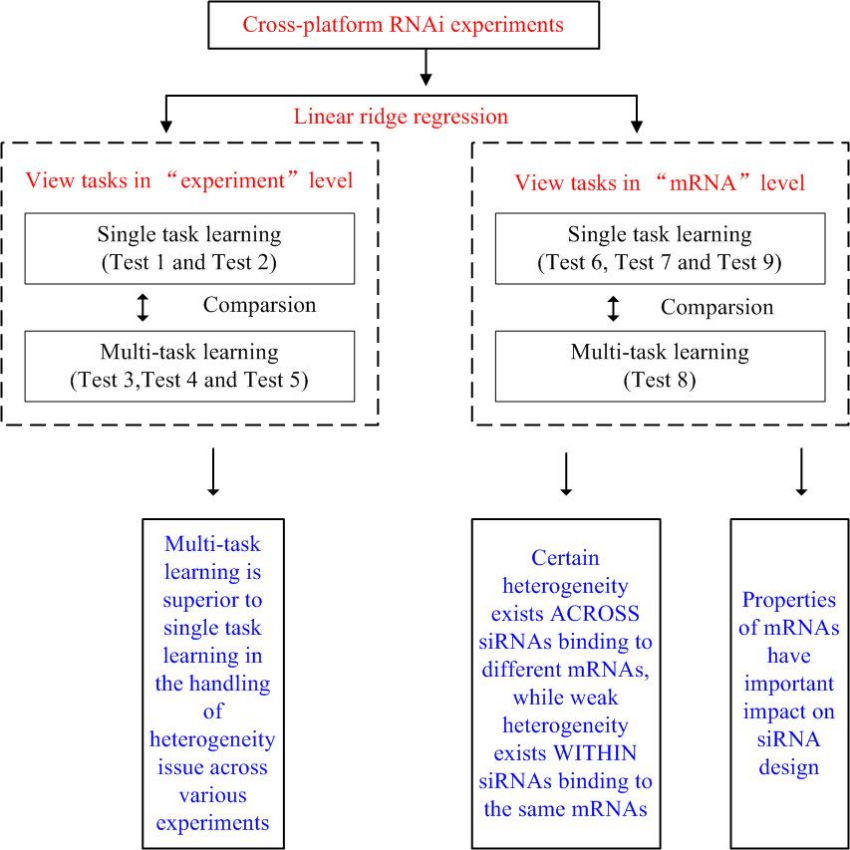
**Computational framework in our study**.

In this study, a comprehensive computational framework for cross-platform RNAi experiment analysis is presented. The workfellow of the framework is shown in Figure 1. Extensive experimental tests were conducted to thoroughly examine the performance of the multi-task learning framework.

#### Algorithm

In this section, we demonstrate how to formulate the cross-platform siRNA efficacy prediction problem as a multi-task learning problem. A critical issue is to learn a set of sparse (regression) functions across the tasks. In particular, *l*1-norm regularization is used to control the number of learned features common for all the tasks, and the whole multi-task learning problem is equivalent to a convex optimization problem [47]. Consequently, the problem is solved iteratively until convergence, by alternately performing an unsupervised step and a supervised step. In the unsupervised step, the common representations shared by the tasks are learned and then in the supervised step, these representations are used to learn the regression functions for each each task. Detailed algorithm derivations can be found in supplementary file [Additional file 1]. A Matlab script package for such multi-task learning in siRNA efficacy prediction is provided, which is accessible freely on our website.

#### Feature selection across tasks

In this section, we show that the proposed multi-task learning provides us an efficient way to evaluate the feature importance in siRNA design across various platforms. Based on the parameter *W *that are derived from Equation (11), the optimal solution for matrix *D *is obtained, which can be used for feature selection. In our case, *D *is a diagonal matrix with *D *= *diag *[λ_1_, ..., λ_*d*_], since *U *is defined as an identity matrix. Specifically, we have(11)

If λ_*i *_≠ 0, the *i*^*th *^feature is the common feature; otherwise, the *i*^*th *^feature is not useful in regression learning across the different tasks, since its regression weights are zeros for all the tasks. The value of λ_*i *_indicates the weight of the corresponding feature, which gives us a quantitative way to evaluate the importance of various features for siRNA design.

## Results and Discussion

In this section, a number of experiments on multi-task learning for cross-platform siRNA efficacy prediction are performed. The siRNA efficacy prediction problem is formulated as a linear ridge regression model and the parameters of this model are tuned with a 5-fold cross-validation process. The root mean square error (RMSE) is adopted as the performance evaluation for different test results. To verify the statistical significance of our model over the baseline algorithms, the paired *t*-test on the experimental results is also conducted [37].

### Multi-task learning for cross-platform siRNA efficacy prediction

#### STUDY 1: Single task learning

In this study, linear ridge regression was we first compared with SVR for single task siRNA efficacy prediction. As an overview, linear ridge regression was shown to achieve the same prediction results as SVR (see Table 3). As a result, linear ridge regression was taken as the chosen learning method in the following study. We show that the 14 cross-platform experiments that we use are indeed have different conditional distribution. We will see that simple combinational or normalization methods only provide very *limited *gain on the improvement of final siRNA efficacy prediction.

**Table 3:** Comparison between linear ridge regression and support vector regression for single task siRNA efficacy prediction.

Test	RMSE						
	
	T1	T2	T3	T4	T5	T6	T7
Linear ridge regression	23.5544	23.0751	12.8477	30.2501	27.8395	32.8025	32.9677
SVR with linear kernel	23.6965	22.1477	13.3903	31.9928	26.1998	32.8823	32.2824
SVR with radial basis function kernel	29.6775	24.4753	13.5664	31.1238	37.2164	36.2681	43.4349

	T8	T9	T10	T11	T12	T13	T14

Linear ridge regression	26.5710	13.6068	13.4394	36.9945	33.6679	17.3333	28.7044
SVR with linear kernel	27.0521	15.2284	25.9767	34.9588	32.8858	19.9620	30.7536
SVR with radial basis function kernel	25.6995	43.3165	25.9767	32.9811	26.6623	19.9620	25.8301

"E" denotes "Experiment". Linear ridge regression and support vector regression(with linear kernel and radial basis function kernel) are trained with 50% of the data from each experiment, respectively. *p*-value calculated by pair *t*-test on linear ridge regression and SVR with linear kernel is 0.2592. *p*-value calculated by pair *t*-test on linear ridge regression and SVR with radial basis function kernel is 0.0913.

In our first test scenario (Test 1), we randomly selected 50% of the data from each experiment(or platform) as the training data to train a linear ridge regression model, and then tested it on the remaining 50% of the data in that experiment. We ran the test 10 times and reported the average RMSE for each experiment. The result of Test 1 was compared with another test scenario (Test 2), in which the same parameters are used under normalization process. In the normalization process, we scaled all the experimental labels (siRNA efficacy values) into [0,1] and pooled 50% of the data from each experiment together to train a general model. Finally, we tested the model on the remaining 50% of the data for each experiment, respectively. The final RMSE was calculated based on the re-scaled predicted and ground-truth labels. Results of these two tests are given in Table 4.

**Table 4:** Single task learning with direct combination and label scaling for siRNA efficacy prediction.

Test	RMSE						
	
	T1	T2	T3	T4	T5	T6	T7
Test 1	23.5500	23.0800	12.8500	30.2500	27.8400	32.8000	32.9700
Test 2	24.9500	29.8900	31.2700	26.8300	32.1900	29.5200	29.2500

	T8	T9	T10	T11	T12	T13	T14

Test 1	26.5700	13.6100	13.4400	36.9900	33.6700	17.3300	28.7000
Test 2	27.2600	15.8700	12.3700	26.2400	30.3800	21.4700	25.9700

"E" denotes "Experiment". Test 1: Selected 50% of the data from each experiment to train a regression model, and tested the model on the remain 50% of the data of each experiment, respectively. Test 2: Scaled all the experimental labels into [0,1] and pooling together 50% of the data from each experiment to train a general model, and tested the model on the remain 50% of the data of each experiment, respectively. *p*-value calculated by pair *t*-test on Test 1 and Test 2 is 0.7043.

From Table 4, we can clearly see that even if the training data labels are scaled to the same level, and the training data are pooled together to train a general model for individual task prediction, the prediction results are still not improving all the time. In fact, we observe worse results in half of the experiments under this general model. Statistical test evaluation on these two models has shown that there is no statistically significant difference between these two prediction results (*p*-value = 0.7043). It indicates that directly scaling the labels and increasing the number of training data by combining the data from cross-platform experiments only provides *limited *help in improving the prediction performance; in many cases the performance is degraded. All tests so far reveal that there exists a high-level of diversity across these 14 experiments, which motivates us to apply more sophisticated multi-task learning in this study.

#### STUDY 2: Multi-task learning

**Figure 2:**
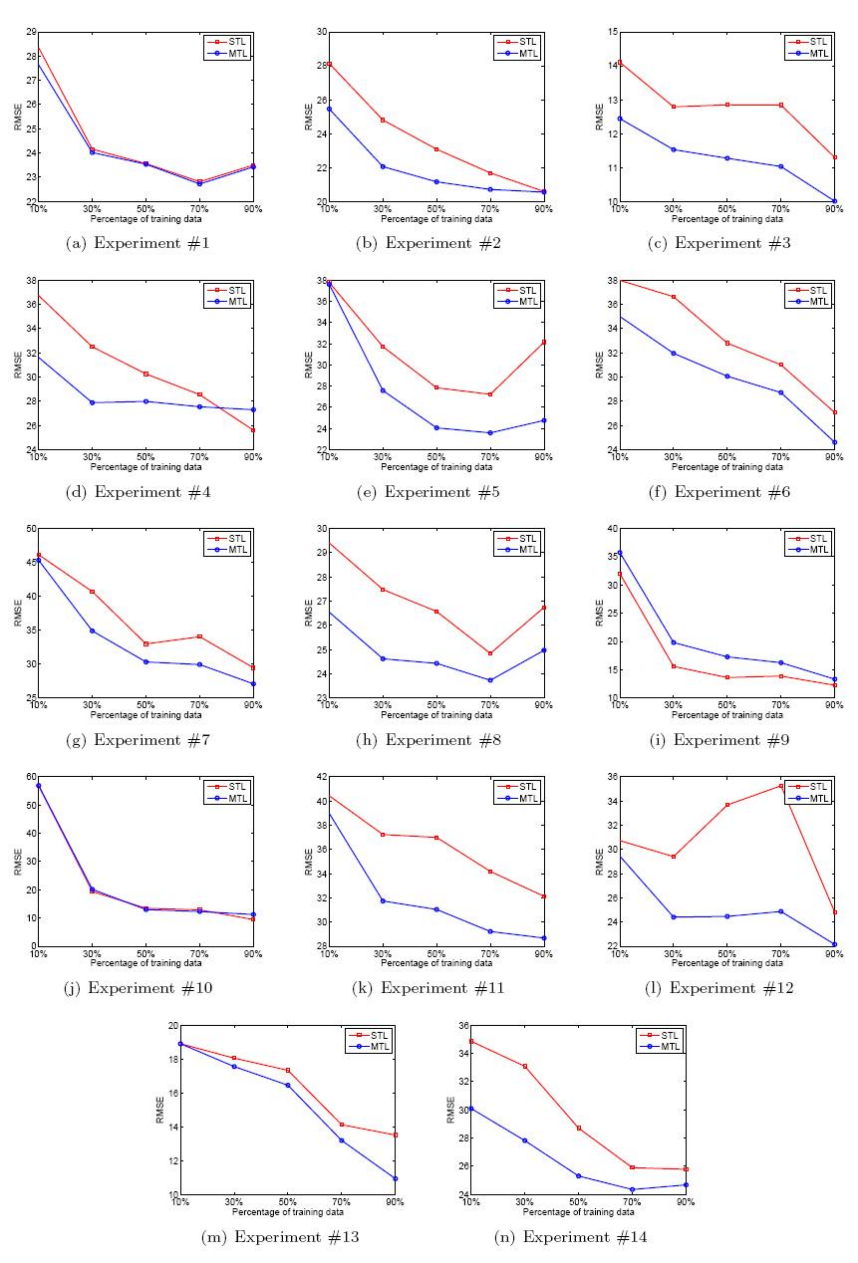
**Comparison between multi-task learning and single task learning for siRNA efficacy prediction**. Each model is trained with 10%, 30%, 50%, 70% and 90% of the data from each experiment, respectively. STL:Single task learning. MTL: Multi-task learning. RMSE: Root mean square error.

In this study, we show that multi-task learning is able to improve the prediction performance as compared to single-task learning. Multi-task learning is performed on the 14 cross-platform experiments with the same setting as Test 1 (50% training data as well as 50% testing data for each experiment). Furthermore, in order to examine the impact of the size of training set on the model's performance, we compared single task learning with multi-task learning trained with other different percentages of data from each experiment. That is, we trained the models with 10%, 30%, 70% and 90% of the whole data, respectively. The testing results are summarized in Table 4 and Figure 2 as Test 3.

From Table 5, it can be clearly seen that multi-task learning achieves better performance as compared to single task learning under various training data percentages for nearly all the experiments. An exception is for experiment 9, in which the two models obtained almost the same level of performance. Pair *t*-test evaluation indicated that multi-task learning is significantly superior to single task learning in siRNA efficacy prediction with all different percentages of training data (*p*-values are listed in Table 5), thanks to the joint learning strategy employed in the multi-task learning model. The prediction performance of most experiments is shown to be correlated to the size of training data, both for single task learning and for multi-task learning, as shown in Figure 2.

**Table 5:** Comparison between multi-task learning and single task learning for siRNA efficacy prediction.

Test 3	RMSE									
	
	Single task learning					Multi-task learning				
	
	10%	30%	50%	70%	90%	10%	30%	50%	70%	90%
E1	28.3515	24.1538	23.5544	22.8080	23.4952	27.6417	24.0150	23.5313	22.7155	23.4194
E2	28.1353	24.7949	23.0751	21.6717	20.5756	25.4531	22.0457	21.1488	20.6969	20.5423
E3	14.1021	12.7868	12.8477	12.8390	11.2925	12.4403	11.5239	11.2708	11.0255	10.0032
E4	36.7345	32.4953	30.2501	28.5389	25.5934	31.6222	27.8789	27.9831	27.5373	27.2947
E5	37.7847	31.7246	27.8395	27.2221	32.1410	37.6029	27.5771	24.0499	23.5798	24.7571
E6	37.9884	36.6409	32.8025	31.0090	27.0574	34.9948	31.9597	30.0650	28.7117	24.6019
E7	46.1408	40.6899	32.9677	34.0303	29.4516	45.3279	34.8915	30.3053	29.9185	27.0738
E8	29.4008	27.4798	26.5710	24.8380	26.7436	26.5423	24.6162	24.4261	23.7297	24.9686
E9	31.9814	15.5796	13.6068	13.8639	12.2373	35.7421	19.8070	17.2665	16.2435	13.3189
E10	56.8917	19.3907	13.4394	12.8776	11.4408	56.8917	19.1463	12.9610	12.2792	11.2242
E11	40.4318	37.2323	36.9945	34.1775	32.1200	38.9771	31.7360	31.0361	29.2156	28.6740
E12	30.7272	29.4070	33.6679	35.2603	24.8004	29.4405	24.4063	24.4616	24.8690	22.1497
E13	18.8997	18.0514	17.3333	14.1208	13.5105	18.8997	17.5524	16.4534	13.1908	10.9338
E14	34.8579	33.0815	28.7044	25.9012	25.7859	30.0917	27.8195	25.3132	24.3546	24.6832

"E" denotes "Experiment". Test 3: Comparison between multi-task learning and single task learning for siRNA efficacy prediction, both trained with 10%, 30%, 50%, 70% and 90% of the data from each experiment, respectively. *p*-values calculated by pair *t*-test on multi-task learning and single task learning with different percentages of training data: 0.0268(10%); 0.0046(30%); 0.0093(50%); 0.0151(70%); 0.0389(90%).

#### STUDY 3: Testing on independent experiments

Another two experiments [23] were also used as independent experiments in this study (Table 1). These experiments were tested in a very low siRNA concentration, including 6 mRNAs with 20 binding siRNAs and 4 mRNAs with 12 binding siRNAs, respectively. Two different tests were performed: (1) Single task learning was compared with multi-task learning on these two independent experiments (Test 4), and (2) Multi-task learning was performed on the two independent experiments together with the former 14 experiments, with a total of 16 experiments (Test 5). Each test kept 50% of the data as a training set as well as 50% of the data as a testing set for each experiment. The average over 10 RMSEs was compared specifically on two independent experiments under two test scenarios. The goal of these tests is to examine the influence of newly added tasks on the existing tasks under our multi-task learning model, and these newly added tasks may be generated in very different experimental conditions. Detailed test results are summarized in Table 6.

**Table 6:** Tests on two independent experiments.

Tests		RMSE	
		
		IE1	IE2
Test 4 (50% training data)	Single task learning	34.1116	35.8600
	
	Multi-task learning	29.7394	30.5459

Test 5 (with added tasks, 50% training data)		26.6910	26.1009

"E" denotes "Experiment". Test 4: Comparison between single task learning and multi-task learning on the two independent experiments, both trained with 50% of the data from each experiment, respectively. Test 5: Multi-task learning on the two independent experiments together with the former 14 experiments, totally 16 experiments, trained with 50% of the data from each experiment, respectively.

We make some observations from the results in Table 6: (1) Multi-task learning gives better performance as compared to single-task learning for the two independent experiments in the siRNA efficacy prediction, and (2) Multi-task learning with more tasks proved to be more helpful for siRNA efficacy prediction, as shown in Test 5. (3) The multi-task regression generalized well to new experimental conditions (and new mRNAs) of the two independent experiments. These conclusions indicate that multi-task learning provides an effective way to alleviate the data insufficiency problem of single task domains by exploiting the available synergy between different tasks. More tasks are expected to provide much more help from a joint learning procedure. Furthermore, with more tasks, multi-task learning can help more to improve the in-silico siRNA design targeted on new mRNAs.

#### Ranking features for cross platform siRNA efficacy prediction

Using our multi-task learning model, we compute the weights for each selected feature in the siRNA efficacy prediction across 14 cross-platform experiments, by considering the learned diagonal matrix *D *calculated in Equation (11). Multi-task learning in this case is also trained with 50% of the data for each experiment and randomly performed by 10 times. The features ranked with their weights are listed in order in Table 2. It can be seen that the position-dependent nucleotide consensus features and Δ G difference between positions 1 and 18 contribute greatly to the design of efficient siRNAs. This conclusion is consistent with the study on the siRNA design as reported in recent works [51,52]. In addition, we can see that the feature of local target mRNA stability has a relatively high weight (0.07) in determining the siRNA efficacy, and this indicates that the properties of mRNA cannot be ignored in the design of potent siRNAs. We will further discuss this issue in the following section.

### Hypothesis: shall we treat task in an "mRNA"-level ?

The impact of mRNA properties (especially the secondary structure of mRNA) on the siRNA binding efficacy has long been a controversial issue [24,52-54]. Traditional studies suggested that it may not be critical to consider the target site's secondary structure in siRNA efficacy prediction. Several models have been presented based on the features merely derived from siRNA sequences to predict their efficacies [18,24]. They show that the mRNA characteristics seem to offer little to the predictive strength of their models. On the other hand, several studies have shown that the properties of mRNA may play an important role in determining the binding efficacy of a siRNA [25,55-57]. These reports motivate us to study the impact of mRNA properties on siRNA binding efficacy from a multi-task learning perspective.

We examine the possibility for siRNA efficacy prediction from a smaller multi-task level, i.e., we consider the task at "mRNA" level instead of the "experiment" level in the efficacy prediction. If the properties of mRNA influence siRNA efficacy, siRNAs that bind to the same mRNA should have some potential connections and thus be viewed as a task in the multi-task learning model. For example, it has been reported that sequence length of target mRNA has certain positive correlation with the activity of binding siRNAs [11]. We speculate that there should exist certain efficacy distribution diversity *across *siRNAs binding to different mRNAs while this efficacy distribution diversity should be weak *within *the siRNAs binding to the same mRNAs. Similar to the tests performed on multiple experiments, combining siRNAs targeted on different mRNAs *may not *benefit the final prediction results. If this is the case, it could be computationally validated that the properties of mRNA indeed have an important impact on the siRNA design.

In order to validate this hypothesis, we performed tests on our siRNA data by grouping the siRNAs binding to 55 mRNAs as 55 tasks. Among them, 20 mRNAs with their number of binding siRNAs in the experiments larger than 5 were selected, and those mRNAs with a very small number of binding siRNAs were removed as they have too few instances to be viewed as a task. Our final dataset includes 20 mRNAs/tasks with a total of 482 siRNA sequences binding to them. A Detailed description of this dataset is given in Table 7.

**Table 7:** Description of the RNAi dataset with viewing each mRNA and its binding siRNAs as a task.

Tasks	#mRNA	#siRNA
T1	M60857	89

T2	U47298	90

T3	J03132	38

T4	U92436	29

T5	LaminA	44

T6	M16553	8

T7	NM_031313	11

T8	NM_020548	9

T9	X75932	10

T10	NM_002046	20

T11	M26071	10

T12	U47298	34

T13	M16553	6

T14	NM_001315	8

T15	NM_000875	16

T16	M25346	8

T17	AF493916	10

T18	AK122643	14

T19	NM_144586	14

T20	M33197	12

"T" denotes "Task".

Similar studies like Test 1 - Test 3 were performed on this dataset, by viewing each mRNA and its binding siRNAs as a task. The new tests are denoted as Test 6 - Test 8 and summarized in Table 8. Table 8 shows that when the tasks were considered in a smaller "mRNA"-level, direct combination and scaling data label still provide *limited *help on the improvement of the prediction performance (*p*-value calculated by pair *t*-test was 0.5862). This indicates that there exists certain efficacy distribution diversity between different tasks. As expected, multi-task learning was superior to single task learning in 17 out of 20 tasks (*p*-value calculated by pair *t*-test was 0.0033).

**Table 8:** Comparison between multi-task learning and single task learning in a "mRNA" task level.

Test	RMSE									
	
	T1	T2	T3	T4	T5	T6	T7	T8	T9	T10
Test 6	22.9156	29.7953	24.4563	20.2755	13.6265	25.5433	28.6792	28.6911	13.8089	47.9704
Test 7	22.0309	28.8772	34.4272	22.4800	29.5645	22.3986	23.4719	42.3385	16.1072	34.2505
Test 8	22.2569	29.4852	22.9905	19.1120	11.7851	23.5123	29.9718	28.4760	11.7036	37.8482

	T11	T12	T13	T14	T15	T16	T17	T18	T19	T20

Test 6	43.6353	13.9306	14.4649	5.6649	35.8113	33.6464	29.6981	29.4559	30.2422	21.0494
Test 7	35.4975	16.8432	13.0795	25.0440	26.3289	36.5158	29.9756	27.0347	26.0495	21.7607
Test 8	41.2163	18.2205	13.6913	5.7872	27.3318	27.5945	23.6955	26.5286	24.3853	16.2990

"T" denotes "Task". Test 6: Selected 50% of the data from each experiment to train a regression model, and tested the model on the remain 50% of the data of each experiment, respectively. Test 7: Scaled all the experimental labels into [0,1] and pooling together 50% of the data from each experiment to train a general model, and tested the model on the remain 50% of the data of each experiment, respectively. Test 8: Multi-task learning for siRNA efficacy prediction, trained with 50% of the data from each experiment, respectively. *p*-value calculated by pair *t*-test on Test 6 and Test 7 is 0.5900. *p*-value calculated by pair *t*-test on Test 6 and Test 8 is 0.0033.

We also designed a test to further examine the data characteristics of the siRNAs *within *one single task. The motivation of this test was discussed previously: since we hypothesized that there exists certain efficacy distribution diversity *across *different mRNAs/tasks in the siRNA efficacy prediction, *little *diversity should exist *within *the task. In this test, two tasks with a large number of siRNA instances were selected as the datasets (Task 1 and Task 2 with 89 and 90 siRNAs respectively). These two datasets (denoted as D1 and D2) are randomly split into 5 sub-tasks and similarly studied as Test 1 - Test 2 are performed on them respectively. Such a study is denoted as Test 9 and summarized in Table 9. It should be noted that for each dataset, since it is selected as a single mRNA with its binding siRNAs, there should be *little *data distribution diversity across the 5 sub-tasks. As shown in Table 9, the data combination and label scaling really work for two datasets in the improvement of efficacy prediction at this time. This is explained by saying that all siRNAs binding to one mRNA are actually homogenous in nature. The prediction performance can thus be improved by increasing the number of homogenous training data.

**Table 9:** Test on the efficacy prediction with siRNAs binding to single mRNA.

	Test 9	RMSE				
		
		Task 1	Task 2	Task 3	Task 4	Task 5
	STL	21.7139	31.3104	22.0464	20.5358	31.3807
	
D1	STL with combination and scaling	20.8203	24.7029	21.2602	18.7345	28.9061

	STL	32.3753	28.3268	27.7405	22.1219	33.1770
	
D2	STL with combination and scaling	26.9951	25.7676	25.0711	19.9418	32.4254

"T" denotes"Task". STL: single task learning. Test 9: Two datasets (D1 and D2) are randomly split into 5 sub-tasks and similar study as Test 1-Test 2 are performed on them respectively.

In conclusion, in siRNA efficacy prediction, there indeed exists certain efficacy distribution diversity *across *the siRNAs binding to different mRNAs, and this distribution diversity seems to be weak *within *the siRNAs binding to the same mRNAs. This result helps validate the observation that the properties of mRNA indeed have influence on potent siRNA design, since certain data heterogeneity has been detected across the siRNAs binding to different mRNAs.

## Conclusions

In this study, a multi-task learning paradigm for cross-platform siRNA efficacy prediction is presented. Extensive empirical tests have been conducted to demonstrate that multi-task learning provides an efficient way for the alleviation of data heterogeneity and insufficiency across multiple tasks. Our method was shown to achieve better prediction performance as compared to the traditional regression models on each individual task independently. This paradigm facilitates different tasks used to learn the hidden data patterns based on a common feature representation. In addition, our experiments validated that siRNA efficacy not only depends on the properties of siRNA, but also on the properties of its targeted mRNA.

Future research on siRNA design could be done to address the data heterogeneity issue further under the multi-task learning scheme. One approach is by taking each mRNA and its binding siRNAs as a task rather than taking each experiment as a task. Another important consideration is to address the issue on finding the major causes for such heterogeneity across different experimental conditions or mRNAs. Our multi-task learning paradigm can only reveal such heterogeneity. For experimental conditions, we wish to further find out what is important on the siRNA concentration, the knockdown assay, etc., in the siRNA design. Similarly, and more importantly, we wish to pursue the question of identifying the most important characteristics that determine the siRNA binding efficacy. Addressing these issues would help to shed new light on why certain genes seem to be easier to be knocked down by RNAi than others. We believe that a better understanding to such problems can be achieved when the amount of available data increases and more new features that influence siRNA-mediated RNA interference are identified.

## Availability

A package of matlab scripts for cross-platform siRNA efficacy prediction under the proposed multi-task learning paradigm is presented. This package together with the datasets used in our manuscript is freely accessible at http://lifecenter.sgst.cn/RNAi/.

## Authors' contributions

QL carried out the design and implementation of the specific computational framework for siRNA efficacy prediction and drafted the manuscript. QX modeled the cross-platform data analysis on biological data as a general multi-task learning scheme. WZ was responsible for the multi-task learning algorithm analysis from a data mining perspective. HX, ZC and QY guided the machine learning study and coordinated the required data analysis. All authors read and approved the final manuscript.

## Appendix - Experimental setting for tests performed in our study

*Test 1 *: For 14 cross-platform experiments as 14 individual tasks, selected 50% of the data from each experiment to train a regression model, and tested the model on the remain 50% of the data of each experiment, respectively.

*Test 2 *: For 14 cross-platform experiments as 14 individual tasks, scaled all the experimental labels into [0,1] and pooling together 50% of the data from each experiment to train a general model, and tested the model on the remain 50% of the data of each experiment, respectively.

*Test 3 *: For 14 cross-platform experiments as 14 individual tasks, perform comparison between multi-task learning and single task learning for siRNA efficacy prediction, both trained with 10%, 30%, 50%, 70% and 90% of the data from each experiment, respectively.

*Test 4 *: For 2 independent experiments, perform comparison between single task learning and multi-task learning on them, both trained with 50% of the data from each experiment, respectively.

*Test 5 *: Multi-task learning on the two independent experiments together with the former 14 experiments, totally 16 experiments, trained with 50% of the data from each experiment, respectively.

*Test 6 *: For the 20 tasks in a "mRNA" level, selected 50% of the data from each experiment to train a regression model, and tested the model on the remain 50% of the data of each experiment, respectively.

*Test 7 *: For the 20 tasks in a "mRNA" level, scaled all the experimental labels into [0,1] and pooling together 50% of the data from each experiment to train a general model, and tested the model on the remain 50% of the data of each experiment, respectively.

*Test 8 *: For the 20 tasks in a "mRNA" level, perform multi-task learning for siRNA efficacy prediction, trained with 50% of the data from each experiment, respectively.

*Test 9 *: Two datasets (D1 and D2) with siRNAs binding to single mRNA are randomly split into 5 sub-tasks and similar study as Test 1-Test 2 are performed on them respectively.

## Supplementary Material

Additional file 1 **Supplementary materials for the manuscript**. This file contains detailed explanation of multi-task learning algorithm, together with the description of the data used in our study Click here for file
