# Supplementary material for: Multi-task learning for cross-platform siRNA efficacy prediction: an in-silico study
Source: BMC Bioinformatics. 2010 Apr 10;11:181. doi: 10.1186/1471-2105-11-181 (PMC2873531; doi:10.1186/1471-2105-11-181)
Supplement: Additional file 1 — Supplementary materials for the manuscript. This file contains detailed explanation of multi-task learning algorithm, together with the description of the data used in our study [file 1471-2105-11-181-S1.DOC]

Multi-task learning for cross-platform siRNA efficacy prediction: an in-silico study

**Supplementary Material**

**Data source descriptions**

The Shabalina et al [1] dataset represents a heterogeneous set of 653 experimentally evaluated 19-nt siRNAs targeted on 52genes from 14 cross-platform experiments. It consists of three subsets of siRNA produced by Dharmacon [2], Amgen Inc [3], Isis Pharmaceuticals [4], and data from other sources [5-13]. All the siRNA sequences are stated in anti-sense format.

The siRNAs in the database were screened for the following criteria. (i) Gene expression was measured after siRNA application relative to untreated control. (ii) At least 5 siRNAs were assayed for a given target mRNA. (iii) The nucleotide sequence of the antisense strand has a perfect complementary target site in the mRNA sequence. Analysis was performed for 19-nucleotide oligonucleotides and did not include the overhangs at the 3'-ends. Please download this dataset from our website:http://ihome.ust.hk/~qiliu/siRNA.html for detailed information.

**Multi-task classification vs. single task classification on Shabalina’s dataset**

It should be noted that our multi-task learning framework can also be used for siRNA efficacy classification based on categorical data. As a complement to the regression model, we just mapped the 14 experiments of Shabalina’s dataset to siRecord [14] to retrieve their categorical labels. Here siRecord is a standardized database to categorize siRNA with consistent efficacy ratings. After such mapping we obtained 9 experiments among the original 14 ones with their corresponding categorical labels. Then based on these data (9 tasks) we built the multi-task classification model vs. single-task classification model to evaluate the classification accuracy defined below:

$$Accuracy=\frac{TP+TN}{TP+FN+TN+FP}$$

where TP is the number of correctly predicted positives, FP is the number of incorrectly predicted positives, TN is the number of correctly predicted negatives and FN is the number of incorrectly predicted negatives.

Noted that although siRecord uses 4 categories to evaluate the siRNA efficacy: *i.e*., *very_high*, *high*, *medium* and *low*, for simplicity we just view the instances with *very_high* and *high* labels as one class and with the other two labels as another class, thus made this multi-class problem to be a traditional binary one. The comparison results are listed in the following table, which indicated that multi-task classification is also significant better than single task learning on this dataset.

**Table: Comparison between multi-task learning and single task learning for siRNA efficacy classification.** (“E” denotes “Experiment”. After mapping Shabalina’s dataset to siRecord, the original 14 experiments were reduced to 9 ones. All tests were trained with 50% of the data of each experiment and predicted on the remain 50% of the data respectively. *p*–value calculated by pair *t*-test on single-task learning and multi-task learning is 0.0087.)

| **Classification** | **Accuracy** | | | | | | | | |
| --- | --- | --- | --- | --- | --- | --- | --- | --- | --- |
|  | **E1** | **E2** | **E3** | **E4** | **E5** | **E6** | **E8** | **E9** | **E10** |
| **Single-task** | 0.7033 | 0.8941 | 0.9545 | 0.5913 | 0.5714 | 0.6385 | 0.7451 | 0.9706 | 0.5250 |
| **Multi-task** | 0.7189 | 0.9088 | 0.9545 | 0.6391 | 0.6714 | 0.7154 | 0.7804 | 0.9824 | 0.6250 |

**Multi-task algorithm**

We presented the detailed multi-task learning algorithm for joint siRNA efficacy prediction. Suppose that we have *T* regression tasks for siRNA efficacy prediction which share a common input feature space . In our case, *T* = 14 for 14 platform experiments, and *d* = 19 for 19 biological features as listed in Table 2 in the manuscript. For task *t*, we are given $n_{t}$ examples , where is the *i^th^ d*-dimensional feature vector and is the corresponding siRNA efficacy value. Note that in practice, the ranges of the siRNA efficacy values are different over the tasks (Table 1 in the manuscript). Based on these data, we aim to jointly estimate *T* regression functions, which approximate the data well and are statistically predictive for the tasks [15].

Formally, we consider the general regression functions $f_{t}$ as


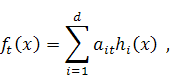


where are the feature mappings to transform the data from the input space to some feature space, and are the regression parameters over the features. Note that the feature mapping functions $h_{i}(x)$ can be designed in a nonlinear form, so that the whole regression task becomes nonlinear. However, in this model, we define the $h_{i}(x)$ as linear forms with $h_{i}(x)=\left\langle U_{i},X\right\rangle$, where with its *i^th^* dimension as 1 and the other dimensions as 0. That is, $h_{i}(x)$ returns the *i^th^* dimension of the input data vector *x*. Hence, the regression functions


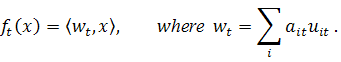


In other words, we define the final regression functions $f_{t}(x)$ as a *linear* form. This is mainly because in our cross-platform siRNA efficacy prediction problem, we not only wish to improve the regression performance on each task but also figure out which input features (i.e. the original 19 biological features as listed in Table 2 in the manuscript) are indeed important across the various platforms.

We denote by *W* the *d* × *T* matrix whose columns are the vectors $w_{t}$, by *U* the *d* × *d* matrix whose columns are $u_{i}$, and by *A* the *d* × *T* matrix with entries $a_{it}$. We then have that *W* = *UA*. In our case, we consider *U* as an identity matrix with *U* = diag [1, 1, .., 1], so *W* = *A*. Our assumption is that the tasks share a small set of features, which means that the matrix *W* has many rows as zero, and thus the corresponding features will not be used for prediction. So we have the optimization as


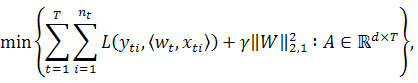


where *L*(·) is the loss function and $\left\|W\right\|_{2,1}^{2}$ is the (2,1)-norm of *W*. Here, an (*r*, *p*) - norm of the matrix *W* is defined as:


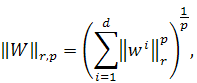


where $w_{i}$ is the *i^th^* row vector of *W*, and its *r*-norm is defined as


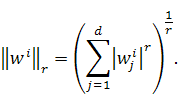


In this equation, our aim in minimizing the first term is to reduce the prediction errors over all the tasks, and our aim in minimizing the second term is to force the learned regression weights to be sparse so that only a small set of features are selected to be shared across the tasks.

The optimization in the above equation is not convex. Fortunately, it can be shown that the optimization can be transformed into an equivalent convex problem [15]. First, for every and (where $S_{+}^{d}$ is the subset of positive semidefinite real symmetric matrices), we define a function:


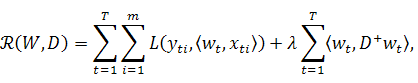


where $D^{+}$ denotes the pseudoinverse of a matrix *D*. Hence, the optimization in the equation is equivalent to:


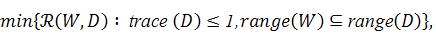


where$trace(D)=\sum_{i=1}^{d}D_{ii}\leq1$; range(*W*) denotes the set { ,for some }. Note that the convex optimization in the this equation can be iteratively solved by alternative optimizing the regression parameters *W* given the matrix *D* and optimizing *D* given *W* [15]. After convergence, we obtained the multi-task regression parameters *W* for further prediction. In our experimental study, we show that the parameter *W* learned by this multi-task learning algorithm can outperform the single task learning approach on siRNA efficacy prediction.

**Appendix - Experimental setting for tests performed in our study**

Test 1 : For 14 cross-platform experiments as 14 individual tasks, we selected 50% of the data from each experiment to train a regression model, and tested the model on the remaining 50% of the data of each experiment, respectively.

Test 2 : For 14 cross-platform experiments as 14 individual tasks, we scaled all the experimental labels into [0,1] and pooling together 50% of the data from each experiment to train a general model, and tested the model on the remaining 50% of the data of each experiment, respectively.

Test 3 : For 14 cross-platform experiments as 14 individual tasks, we performed comparison between multi-task learning and single task learning for siRNA efficacy prediction, both trained with 10%, 30%, 50%, 70% and 90% of the data from each experiment, respectively.

Test 4 : For 2 independent experiments, we performed comparison between single task learning and multi-task learning on them, both trained with 50% of the data from each experiment, respectively.

Test 5 : Multi-task learning on the two independent experiments together with the former 14 experiments, totally 16 experiments, trained with 50% of the data from each experiment, respectively.

Test 6 : For the 20 tasks in a “mRNA" level, we selected 50% of the data from each experiment to train a regression model, and tested the model on the remaining 50% of the data of each experiment, respectively.

Test 7 : For the 20 tasks in a “mRNA" level, we scaled all the experimental labels into [0,1] and pooling together 50% of the data from each experiment to train a general model, and tested the model on the remaining 50% of the data of each experiment, respectively.

Test 8 : For the 20 tasks in a “mRNA" level, we performed multi-task learning for siRNA efficacy prediction, trained with 50% data from each experiment, respectively.

Test 9 : Two datasets (D1 and D2) with siRNAs binding to single mRNA are randomly split into 5 sub-tasks and similar studies as Test 1-Test 2 are performed on them respectively.

# **References**

[1]. Shabalina,S.A. *et al*. (2006) Computational models with thermodynamic and composition features improve siRNA design. *BMC Bioinformatics*, **7**, 65.

[2]. Khvorova,A. *et al*. (2003) Functional siRNAs and miRNAs exhibit strand bias. *Cell*,**115**, 209–216

[3]. Reynolds,A. *et al*. (2004) Rational siRNA design for RNA interference. *Nat. Biotech.*,**22**, 326–330.

[4]. Vickers,T.A. *et al*. (2003) Efficient reduction of target RNAs by small interfering

RNA and RNase H-dependent antisense agents. *J. Biol. Chem.*, **278**,7108–7118.

[5]. Aza-Blanc,P. *et al*. (2003) Identification of modulators of TRAIL-induced apoptosis via RNAi-based phenotypic screening. *Mol. Cell*, **12**, 627–637.

[6]. Harboth,J. *et al* (2003) Sequence, chemical and structural variation of small interfering RNA sans short hairpin RNAs and the effect on mammalian gene silencing. *Antisense Nucleic Acid Drug Dev.*, **13**, 83–105.

[7]. Holen,T. *et al*. (2002) Positional effects of short interfering RNAs targeting the human coagulation trigger Tissue Factor. *Nucleic Acids Res*., **30**, 1757–1766.

[8]. Kumar,R. *et al*. (2003) High-throughput selection of effective RNAi probes for gene silencing. *Genome Res.*, **13**, 2333–2340.

[9]. Hsieh,A.C. *et al*. (2004) A library of siRNA duplexes targeting the phosphoinositide 3-kinase pathway: determinants of gene silencing for use in cell-based screens. *Nucleic Acids Res.,* **32**, 893–901.

[10]. Ui-Tei,K. *et al.* (2004) Guidelines for the selection of highly effective siRNA sequences for mammalian and chick RNA intereference. *Nucleic Acids Res.*, **32**, 936–948.

[11]. Jackson,A.L. *et al.* (2003) Expression profiling reveals off-target gene regulation by RNAi. *Nat. Biotech*., **21**, 635–637.

[12]. Giddings,M.C. *et al*. (2000) ODNBase—a web database for antisense oligonucleotide effectiveness studies. *Bioinformatics*, **16**, 843–844.

[13]. Kawasaki,H. *et al*. (2003) siRNAs generated by recombinant human dicer induce specific and significant but target site-independent gene silencing in human cells. *Nucleic Acids Res*., **31**, 981–987.

[14]. Ren Y, Gong W, Zhou H,Wang Y, Xiao F, Li T: siRecords: a database of mammalian RNAi experiments and efficacies. *Nucleic Acids Research* 2009, **37**(Database issue):D146.

[15]. Argyriou A, Evgeniou T, Pontil M: Multi-task feature learning. In *Advances in Neural Information Processing Systems: Proceedings of the 2006 Conference*, MIT Press 2007:**41**.
